# Validation of the least squares fitting method (lsf) during nava and psv ventilation

**DOI:** 10.1186/2197-425X-3-S1-A1005

**Published:** 2015-10-01

**Authors:** F Dalla Corte, S Spadaro, S Grasso, V Cricca, G Biondi, A Fogagnolo, G Valpiani, R Di Mussi, S Bertacchini, MV Colamussi, E Marangoni, CA Volta

**Affiliations:** Morphology, Surgery and Experimental Medicine, University of Ferrara, Ferrara, Italy; Emergency and Organ Transplantations, University of Bari, Bari, Italy

## Introduction

The Least Squares Fitting (LSF) is a computerized method of analysis of respiratory system mechanics. It is based on applying a regression analysis for every sample points of the loop of pressure, flow and volume by fitting the equation **P**_**aw**_**= R**_**rs**_**× V' + V**_**T**_**/C**_**rs**_**+ PEEP**_**tot**_ during inspiration [1]. This technique has been already validated in Controlled Mechanical Ventilation (CMV) and at high level of Pressure Support Ventilation (PSV) [2]. However this method gives unreliable values of resistance (R_rs_) and elastance (E_rs_) in presence of inspiratory muscle activity and in absence of an adequate neuromuscular coupling. We reasoned that NAVA (Neurally-Adjusted Ventilatory Assist) ventilation should assure a better neuromuscular coupling compared to PSV and hence the coefficient of determination (CD) of the above equation should be much higher during NAVA ventilation.

## Objectives

The aim of this study was to prove the efficacy of the LSF method in obtaining reliable respiratory mechanics data in two different ventilatory modes.

## Methods

Twelve patients with acute respiratory failure were enrolled at the admission to the ICU and ventilated using in random order either PSV or NAVA for 3 hours with the same Positive End Expiratory Pressure (PEEPe) and tidal volume (VT) settings. Flow and pressure traces were recorded and subsequently analyzed using the LSF method to obtain data of Rrs, Ers, PEEPtot and coefficient of determination (CD). NAVA and PSV were first compared in terms of CD during the 3 hours of recording. Furthermore, we selected 100 consecutive breaths for each patient in each ventilatory mode to compare the values of elastance (the only non-flow dependent of the equation of motion) obtained either in NAVA or PSV.

## Results

**Figure 1:**
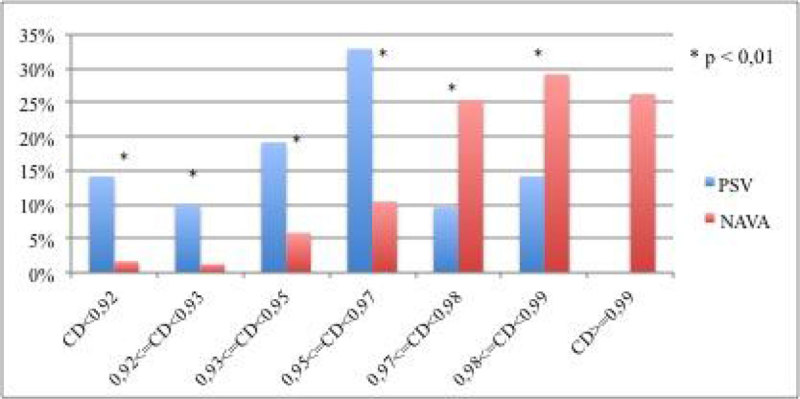
Figure 1

**Figure 2:**
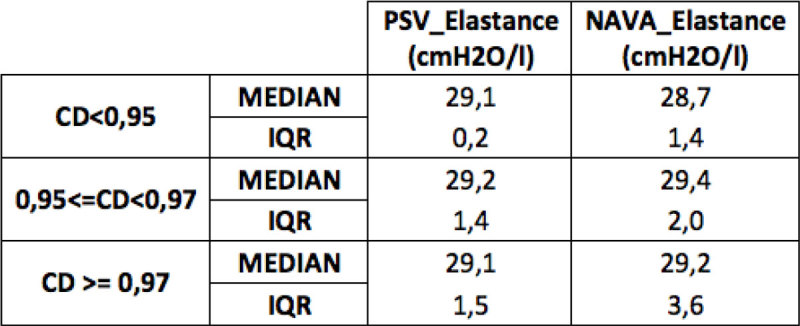
Figure 1

The CD during NAVA ventilation was statistically higher than that obtained during PSV (Figure 1) (Chi-squared test: p < 0.001). CD intervals are based on the percentiles of CD distribution in the two ventilatory modes. E_rs_ values for PSV and NAVA are presented in Figure 2.

## Conclusions

Our results seem to confirm that the neuromuscular coupling is much better preserved during NAVA than during PSV.
